# Pupil dilation reflects the authenticity of received nonverbal vocalizations

**DOI:** 10.1038/s41598-021-83070-x

**Published:** 2021-02-12

**Authors:** Gonçalo Cosme, Pedro J. Rosa, César F. Lima, Vânia Tavares, Sophie Scott, Sinead Chen, Thomas D. W. Wilcockson, Trevor J. Crawford, Diana Prata

**Affiliations:** 1 Instituto de Biofísica e Engenharia Biomédica, Faculdade de Ciências da Universidade de Lisboa, Campo Grande, 1749-016 Lisboa, Portugal; 2 HEI-LAB: Human-Environment Interaction Lab, Universidade Lusófona de Humanidades e Tecnologias, Campo Grande, 376, 1749-024 Lisboa, Portugal; 3 Instituto Universitário de Lisboa (Iscte-IUL), CIS-Iscte, Avenida das Forças Armadas, 1649-026 Lisboa, Portugal; 4 Faculdade de Medicina da Universidade de Lisboa, Avenida Professor Egas Moniz MB, 1649-028 Lisboa, Portugal; 5 Institute of Cognitive Neuroscience, University College London, Alexandra House, 17-19 Queen Square, Bloomsbury, London, UK; 6 Risk Society and Policy Research Center, National Taiwan University, Roosevelt Rd., Daan Dist., Taipei, 10617 Taiwan; 7 Department of Psychology, Lancaster University, Lancaster, LA1 4YF UK; 8 School of Sport, Exercise, and Health Sciences, Loughborough University, Clyde Williams Building, Epinal Way, Loughborough, LE11 3GE UK; 9 Department of Neuroimaging, Institute of Psychiatry, Psychology and Neuroscience, King’s College London, 16 De Crespigny Park, Camberwell, London, SE5 8AF UK

**Keywords:** Cognitive neuroscience, Emotion, Social behaviour, Empathy, Human behaviour

## Abstract

The ability to infer the authenticity of other’s emotional expressions is a social cognitive process taking place in all human interactions. Although the neurocognitive correlates of authenticity recognition have been probed, its potential recruitment of the peripheral autonomic nervous system is not known. In this work, we asked participants to rate the authenticity of authentic and acted laughs and cries, while simultaneously recording their pupil size, taken as proxy of cognitive effort and arousal. We report, for the first time, that acted laughs elicited higher pupil dilation than authentic ones and, reversely, authentic cries elicited higher pupil dilation than acted ones. We tentatively suggest the lack of authenticity in others’ laughs elicits increased pupil dilation through demanding higher cognitive effort; and that, reversely, authenticity in cries increases pupil dilation, through eliciting higher emotional arousal. We also show authentic vocalizations and laughs (i.e. main effects of authenticity and emotion) to be perceived as more authentic, arousing and contagious than acted vocalizations and cries, respectively. In conclusion, we show new evidence that the recognition of emotional authenticity can be manifested at the level of the autonomic nervous system in humans. Notwithstanding, given its novelty, further independent research is warranted to ascertain its psychological meaning.

## Introduction

We express emotions in social interactions to convey information about our affective states and intentions, which is essential for communication. In turn, we constantly evaluate the authenticity behind others’ emotional expressions, even involuntarily. This complex process of cognitive empathy (also known as theory of mind or mentalization), uses perceptual and sensorimotor cues^1^, and allows for an adequate social response, from more intuitive to more deliberate, such as decisions to trust or not to trust in the other individual, and thus whether to cooperate or to compete^2^. Given that these decisions are vital for social bonding, defense from aggression, and ultimately social network structure, they have been of utmost importance for human survival^3^. As such they may be hard-wired in our nervous system. However, it is still unknown whether and how the evaluation of the authenticity of another’s emotional expression engages the autonomic nervous system of the person perceiving it.

Emotions can be effectively expressed vocally without semantic content, such as in laughter and crying, unconstrained by linguistic structures^4–6^. Even in the absence of a situational context, nonverbal vocalizations provide relevant cues to infer emotional states^7^, and their recognition can transcend cultures^8^. Nonverbal vocalizations can vary in emotional category (e.g., amusement, sadness, anger, fear, surprise and disgust)^9^, valence^4^, arousal^4^, affiliative value (i.e. emotional contagion)^10^ and authenticity^1^. Crying, for example, is an intense emotional expression of a negative state often accompanied by lacrimation, which, in a social context, is assumed to have the purpose of eliciting help from listeners^11^, or, in an interpersonal context, is understood to function as relief and improve mood after shed tears^12^. Conversely, and vastly more studied in nonverbal vocalization literature, laughter is an emotional expression of a positive state and has the role of promoting and maintaining social bonding^13^. The underlying emotion causing the expression of crying or laughter may vary such that acoustic differences have been found for different kinds of laughter (e.g. ticklish versus emotional)^14,15^, for example.

Correctly recognizing the authenticity of others’ laughter or crying is a social skill essential for avoiding deception^2,16^. Authenticity recognition is the ability of discerning between an authentic (genuine) versus an acted (deliberate) emotional expression, for which we use acoustic differences in the case of nonverbal vocalizations^15,17^. We have shown that authentic laughter and cries are often more highly pitched, longer in duration and have different spectral characteristics compared to their acted variants^18^. Additionally, across various emotions, higher and more variable pitch, lower harmonicity, and less regular temporal structure are the best predictors of authenticity judgements^19^. Listeners recognize authenticity in laughter at roughly 70% accuracy (67%^17^, and, as we have shown, 72%^15^ and 63%^19^). Furthermore, authentic laughter is rated as more arousing and more positive than acted laughter^15^. Generally, genuine emotional expressions are produced reactively while deliberate expressions are intentional and controlled forms of communication^13^. Whereas authentic laughter is genuine and usually an immediate reaction to a positive and surprising stimulus, acted laughter is associated with polite agreement and (real or fake) appreciation^13,17^. While authentic crying is also genuine and usually negative stimulus-driven, acted crying is associated with (manipulative) social deception^16^.

We have shown, during passive listening, the anterior medial prefrontal cortex (amPFC) and anterior cingulate cortex to be more strongly engaged for acted (than authentic) laughter^1^. This is consistent with the view that interpreting non-authentic stimuli, and solving its ambiguity, is relatively more cognitively demanding^20^, engaging cognitive empathy (i.e. mentalization) to a higher degree. In addition, a linear decrease in amPFC activation as perceived authenticity increases^18^, has been found. To our knowledge, the electrophysiological response to nonverbal vocalization authenticity has not yet been investigated, but visual stimuli’s authenticity (e.g. genuine vs. ambiguous smiles) has been reflected in early event related potentials (ERPs) components’ amplitude, e.g. P200, albeit only when the ambiguous smiles were blended with angry eyes, suggesting it is processed very early, and dependent on the salience of the expression^21^. More evidence that cognitive strategies are required to infer authenticity and that these demand a level of social maturity and experience, comes from the findings that adults’, but not children’s, cognitive and emotional empathy scores correlated with authenticity discrimination of happy faces^22^. In addition, high *emotional* trait empathy may also help authenticity recognition by facilitating the simulation of the emotion itself (through emotional contagion, i.e. the propensity to resonate with others’ emotions), leading to a stronger emotional response to authentic expressions^22^. Indeed, we have reported that emotional trait empathy, emotional contagion and authenticity recognition in laughter to be positively associated^10^.

Pupil size is used as a proxy of both arousal^23^ and cognitive effort in emotion research^24,25^ and it depends on autonomic peripheral nervous system activity, which may in turn be elicited by central nervous system input. Activation of the iris dilator muscle^26^ stems from a sympathetic response (known in the context of the ‘fight-or-flight’ mode) triggered by adrenaline release, and produces an enlargement of the pupil size (i.e. pupil dilation). Activation of the iris sphincter muscle^26^ stems from a parasympathetic response (typical of the ‘rest and digest’ mode)^23,27^, and produces a reduction of the pupil size (i.e. pupil constriction). The pupil dilates with higher arousal elicited by a stimulus^23^, thus, emotionally charged vocalizations evoke higher pupil dilation compared to neutral ones (with differences between positive and negative showing mixed results^28^). In addition, the pupil also dilates with cognitive effort^29,30^, and has been associated with amPFC activity, a key area for cognitive empathy as abovementioned^31^. Furthermore, pupil mimicry (i.e. synchronization) during social interaction is proposed to be an emotional contagion mechanism and an implicit form of social communication^32^. Indeed, pupil size changes are elicited by emotional contagion^32^ which in turn facilitates authenticity recognition at least for laughter^10^.

Examining how sensitive is, if at all, pupil size to the authenticity of perceived emotional expressions can reveal underlying processes of authenticity discrimination; in particular, whether the autonomic system is involved. Furthermore, by combining this examination with empathy traits and behavioural measurements, one could disentangle to what extent authenticity discrimination is a cognitively demanding and/or affective process. However, the direct association between pupil size and the recognition of authenticity has not yet been examined, to our knowledge. Although authenticity recognition is an essential cognitive empathy skill for an adaptive social behavior, and its central nervous system correlates have started to be unraveled, it is still unknown whether it engages the peripheric autonomic nervous system. Herein, we asked for the first time, whether the authenticity of an emotional expression induced an autonomic nervous system response during its perception. In the present study, we tested this by assessing the effect of nonverbal vocalizations’ authenticity on the pupil size of the listener. We asked participants to rate the degree of authenticity in authentic and acted laughs and cries, during which we recorded changes in their pupil size and, posteriorly, the degree of emotional contagion and arousal of the same stimuli. Lastly, we measured the participants’ cognitive and emotional empathy traits. Given the unprecedented examination into the autonomic nervous system’s activity during authentic discrimination and the dual-proxy nature of pupil response, we had two possible (directional) predictions alternative to the null hypothesis, one assuming a preponderance of arousal, the other of cognitive effort: (1) authentic vocalizations would elicit *higher* pupil dilation compared to acted, because they have been found to be more arousing in general^19,33^, and pupil dilation increases with arousal^23^; or (2) by the contrary, authentic vocalizations would elicit *lower* pupil dilation, because authenticity discrimination, at least in laughter, has been found to decrease the engagement of prefrontal cognitive empathy-relevant brain areas^1,18^, suggesting lower cognitive demand. As such, we aimed to disentangle whether it is arousal (supposedly higher in authentic) or cognitive load (supposedly higher in acted vocalization), during authenticity recognition, that engages autonomic nervous system the most. Additionally, by including an examination of the neural correlates of crying for the first time, we explored how our two predictions would depend on emotion valence (i.e. would differ between laughs and cries); which is warranted given that the social meaning of authenticity in each emotion can be quite different (i.e. a fake laugh can signify benign politeness or sarcasm, but a fake cry can mean costly deceit). Complementarily, (1) given that authenticity discrimination has been positively correlated with cognitive^22^ and emotional empathy^10,22^, we test the association of these empathy traits with our behavioral and pupil size measures; and (2) we also ask whether previous positive associations between perceived authenticity and arousal^18^ and, for the first time, emotional contagion^1,10^, in laughter, extend to crying.

## Materials and methods

### Participants

All methods were carried out in accordance with relevant guidelines and regulations. Thirty-eight individuals were recruited to participate in the experiment via the laboratory’s recruitment website. Eight participants were excluded from analysis due to technical problems in data acquisition (i.e. no eye data recorded across session or task trigger misconfiguration), and 2 due to inadvertently uncontrolled room luminosity, thus the final sample consisted of 28 participants (13 male and 15 female) with an average age of 23.0 years (SD = 1.38, ranging from 21 to 26 years old). The inclusion criteria were right handedness (Edinburgh Handedness Inventory; due to EEG measures being collected in the same study)^34^ and European Portuguese as first language. For female participants, an additional inclusion criterion was to be on the active weeks of the contraceptive pill, as time of the menstrual cycle has been shown to affect emotion recognition task performance^35^. Participants provided written informed consent and were paid for their participation. To ascertain a normal distribution in terms of working memory, emotional state, and a mentally healthy sample, the Positive and Negative Affect Schedule (PANAS)^36,37^ (Positive Affect Score—M = 43.41, SD = 8.52; Negative Affect Score—M = 29.95, SD = 9.80); the Forward and Backward Digit Span Tests of Working Memory Index of the Weschler Adult Intelligence Scale—Third Edition (WAIS—III)^38^ (WM Index = 19.71, SD = 3.66); and the Brief Symptom Inventory was administered (Global Severity Index (GSI)—M = 0.66, SD = 0.49), respectively.

### Stimuli

The set of auditory stimuli comprised authentic and acted nonverbal vocalizations of amusement—laughter—and of sadness—crying—along with neutral vocalizations (e.g. the vowel ‘ah’ uttered with neutral intonation)^39^. The neutral vocalizations were included only for comparison against cries and laughs; there were no acted-neutral or authentic-neutral stimuli, as neutral stimuli are, by nature, not affective and thus cannot be authentic or acted. Authentic vocalizations were elicited by the speakers while watching humorous videos (authentic laughter) or while recalling truly upsetting events (authentic crying), whereas acted laughter and crying were acted under full voluntary control. We used vocalizations we previously validated at the behavioral and neuroimaging level^1,40^, as follows in short. Three male and 3 female speakers recorded the stimuli in an anechoic chamber. For authentic laughter, YouTube videoclips which were previously identified by the speakers as humorous, were shown to induce them to laugh out loud. For authentic crying, speakers were encouraged to recall personal upsetting events and/or start by posing crying in order to transition genuine crying. Lastly, the speakers were asked to simulate acted laughter and crying without feeling any genuine amusement or sadness, respectively. To avoid carry-over effects of genuine amusement or sadness, the recording of acted laughter or crying always preceded the recording of authentic laughter or crying. From each recording session, separate audio files were created for laughter and crying, sampled at 44.1 kHz to mono.wav files with 16-bit resolution. To control for variability in the acoustic properties of the sounds, the audio was normalized for root-mean-square (RMS) amplitude using Praat software (www.praat.org)^40^. In this study, for each condition (authentic laughter, acted laughter, authentic crying, acted crying), 18 vocalizations were used and presented twice. An additional 60 neutral vocalizations were presented once. In the end, the stimuli set consisted of 132 vocalizations, each with different durations (in milliseconds—authentic laughs: M = 2399.94, SD = 460.73, range = 1536.00, 3141.00; acted laughs: M = 2248.89, SD = 400.15, range = 1710.00, 2903.00; authentic cries: M = 2684.55, SD = 289.36, range = 2079.00, 2993.00; acted cries: M = 2322.11, SD = 351.48, range = 1959.00, 2990.00; neutrals: M = 2498.74, SD = 292.08, range = 2057.00, 2930.00). The acoustic properties of the stimuli (duration (ms), mean fundamental frequency—F(0), mean intensity (dB)) were obtained using Praat software and reported in more detail in supplemental material (Supplementary Table S1).

To compare the acoustic properties between conditions, the main effects of authenticity and emotion were tested using non-parametric Kruskal–Wallis H Tests (due to the normality assumption not being fulfilled for their mean in each condition) and reporting ε^2^ for effect size for duration, intensity and fundamental frequency. Further, pairwise comparisons were performed. There was an association of authenticity with pitch (H(1) = 16.53, *p* < 0.001, ε^2^ = 0.97) and duration (H(1) = 9.98, *p* = 0.002, ε^2^ = 0.59), whereby authentic vocalizations had higher pitch and were longer than acted ones; but not with intensity (H(1) = 0.28, *p* = 0.596, ε^2^ = 0.02). Additionally, there was an association of emotion with pitch (H(1) = 63.81, *p* < 0.001 , ε^2^ = 3.75), whereby negative vocalizations had higher pitch than positive and neutral, and positive more than neutral; and intensity (H(1) = 62.59, *p* < 0.001, ε^2^ = 3.68) where positive vocalizations had higher intensity than negative and neutral; but not with duration (H(1) = 2.57, *p* = 0.227, ε^2^ = 0.15).

### Task

Before the start of the task, which included concomitant pupil size recording (described below), the participants were informed that they would listen to sounds, and that they would be required to rate the sounds in terms of their perceived authenticity. For neutral sounds, the participants were instructed to just attend to the stimulus. Always showing a fixation cross on screen, a trial started with silence for 4000 ms plus a jitter of 500 ms, followed by the presentation of the sound stimuli and then a 3000 ms interstimulus interval. After this, a 7-point Likert scale showed on screen for up to 5000 ms for the participants to answer their perceived authenticity ranging from 1 (“Genuine” —authentic) to 7 (“Posed”—acted). For ease of interpretation and discussion, the scale was reversed for the statistical analysis. The task took 36 min to complete and had 204 trials in a pseudo-randomized and fixed sequence, balancing condition transitions trial-by-trial so that conditions transitioned equally between themselves as to minimize the effects of pupil habituation^41^.

After the above pupillometry-recorded task round, participants (n = 21 of the 28, due to unforeseen time limitation) were instructed to evaluate the perceived arousal and emotional contagion of the previously presented vocal stimuli in a 7-point Likert scale (Arousal: 1- Low arousal, 7- High arousal; Emotional contagion: 1- Not contagious at all; 7- Highly contagious), except for neutral sounds. Divided in two blocks, first they rated all 72 emotional sounds in terms of their arousal (72 sounds in total, 18 for each condition: authentic laughter, acted laughter, authentic crying, acted crying), and in the second block they rated the same sounds for their contagion. Herein, a trial consisted in 1500 ms plus 500 ms of jitter, then stimuli presentation followed by 1000 ms of interstimulus interval, always with a fixation cross on the screen, after which the Likert scale of arousal or contagion (depending of the block) would be shown. This task lasted for 15 min and had 124 trials, also pseudo-randomized and fixed sequence with balanced condition transitions. Herein, each stimulus was presented once in each block.

### Pupil size recording

The fixation cross and Likert scales were shown in a Lenovo 23.8-inch screen with 1920 × 1080 resolution and 60 Hz refresh rate. Gaze tracking and pupil measurements were recorded using the SR Research EyeLink 1000 Plus eye tracker. A chin rest was used to minimize head movement and keep a fixed distance to both the screen and camera, at approximately 56 cm for all participants. Raw data was collected monocularly at 1000 Hz with average accuracy of 0.15 visual angle.

After data collection, the pupil size was down sampled to 250 Hz (to save on computational costs), blinks and datapoints 100 ms before and after blinks were considered as missing data. A low-pass filter 4 Hz cut-off frequency was applied to the signal. Pre-trial baseline was obtained for each trial as the median pupil size immediately before stimuli onset, in an interval that was 2% of the whole trial, which varied depending on the duration of the stimuli (M = 204.14, SD = 4.71 ms). This median value was then subtracted across all datapoints of its trial as advised in the literature^42^. Finally, if the missing data did not exceed 600 ms, as blinks longer than this are considered microsleeps^43–45^, the signal was linearly interpolated^46,47^. These preprocessing steps were employed for all pupil dilation analyses. Four time windows of 1 s, after stimuli onset, were created to evaluate pupil size measures across time. As our stimuli had variable duration, a 4-s analysis period ensures the inclusion of peak dilation and consequent return to baseline, so that both peak and mean pupil diameter can be adequately measured. The segmentation into time windows allowed a more sensitive and thorough assessment of pupil response to authenticity and emotion, and its consequent constriction during and post stimuli presentation, and has been precedently employed^48,49^. For each trial, maximum and mean pupil sizes were extracted in each individual time window. Lastly, as recommended in guidelines for pupillometry pre-processing and analysis^50^, and common^31,51,52^, we have excluded from the group analysis maximum and mean outlier pupil size datapoints at the level of the trial. Datapoints were considered outliers if their mean per condition was above or below 1.5 times the interquartile range (following standard criteria^53^).

### Procedure

Each participant underwent the experiment in one session lasting 2 h and a half, sitting comfortably in a quiet room at the Centre for Clinical Research (Centro de Investigação Clínica) of the Medical Academic Centre of Lisbon (Centro Académico Médico de Lisboa), whose Ethical Committee approved all experimental protocols. During the task, the auditory stimuli was presented binaurally through a set of Senheiser CX 3.00 ear-canal phones at a comfortable listening level that was individually adjusted at the start of the experiment. The experiment was developed using MATLAB version 8.3.0 (R2014a) with Psychtoolbox 3^54^. Participants were encouraged to respond as intuitively as possible. Buttons of the response pad were marked with the Likert scale points to minimize memory demands. To facilitate the response, participants were asked to put three fingers of their left hand in response keys 1, 2 and 3 and four fingers in the remaining response keys. Three pauses of 30 s were distributed equally along the experiment to minimize fatigue. Concomitant electroencephalography recording also took place (data not yet analyzed). After the pupillometry-recorded task, participants rated their perceived arousal and emotional contagion for every sound, and finally responded to the Empathy Quotient (EQ) and the Reading the Mind in the Eyes Test (RMET), which assesses emotional and cognitive trait empathy.

### Statistical analysis

Correlation analyses (between trial-by-trial stimuli and pupillometry measures; between trait empathy scores and pupillometry measures, with authenticity as a categorical moderator; and between trait empathy scores and authenticity discrimination index—described below) were performed in R software 3.6^55^, and when applicable, using the rmcorr package^56^. To verify reliability of the rating scales, Cronbach’s alpha was calculated for each one^57^. To infer the main effects of authenticity and its interaction with emotion on each behavioral measure (authenticity, arousal and emotional contagion ratings), a repeated measure Analysis Of Variance (rpANOVA) model for each measure was conducted in SPSS (IBM Corp. Released 2017, IBM SPSS Statistics for Windows, Version 26.0. Armonk, NY: IBM Corp.). For completeness, the association of authenticity, arousal and contagion ratings with the two pupil size measures was also estimated. For authenticity discrimination, ratings between 1 and 3 were converted to ‘posed’, and ratings between 5 and 7 were converted to ‘authentic’. For completeness and to attempt a replication of our previous work (Neves et al., 2018), we also computed an index for authenticity detection ability, for each emotion and each subject, by subtracting the average authenticity ratings of acted stimuli from the average authenticity ratings of authentic ones; and we report, in supplemental material (Supplementary Table S2), the correlation between these indexes and individual trait empathy (EQ and RMET questionnaire) scores.

Pupillometry-wise, two separate rmMANOVA models were constructed in SPSS, each including Time window as a within-subject factor (0–1, 1–2, 2–3, 3–4 s): one to estimate the main effect of the within-subject factor Authenticity (authentic, acted) and its interaction with the within-subject factor Emotion (laughter, crying); and another to estimate the main effect of emotion (laughter, neutral, crying) given the inexistence of authentic neutral and acted neutral sounds. The dependent variables of both rmMANOVAs were the two pupil size measures (maximum and mean pupil size) for which we report the two corresponding univariate rmANOVA results. All effects of interest were followed up by post hoc pairwise comparisons and reported after False Discovery Rate (FDR) correction in R^55^ (and considered statistically significant when FDR corrected *p* < 0.05). Partial Eta-square (*η*_*p*_^2^) is reported as a measure of effect size. We report the main effect of authenticity and emotion as well as interactions between them and with time window. Little’s Missing Completely At Random (MCAR)^58^ tests were performed for all pupil size measures to test the randomness of missing values. Given our previous work suggesting the acoustic properties of the stimuli mediate authenticity recognition^19^ and since they naturally differ depending on their authenticity and/or emotion, complementary repeated measures correlation analyses between each stimuli (trial-by-trial) and pupil size measures were conducted to evaluate their direct effect on pupil dilation and hence their potentially mediating role^59^ (see Results as supplementary material (Supplementary Table S5)).

### Power

To our knowledge, only two studies^60,61^, using affective auditory stimuli, have reported effects sizes of the effect of emotion on pupil size, whilst none have reported effects of authenticity. One^60^ used 26 participants to report a main effect of emotion (positive, negative and neutral) on the mean gradient of pupil diameter, across 0–2 s after stimuli onset, with a size of *η*_*p*_^2^ = 0.20, while the other^61^ used 97 subjects to report a main effect of pleasantness (pleasant, unpleasant and neutral) on pupil diameter with a similar effect size of *η*_*p*_^2^ = 0.22 for the peak time window (2—4 s). We note these results were not independent as the latter study has a 33% stimuli overlap with the former study. An a priori power analysis, considering *η*_*p*_^2^ = 0.20, in GPower 3.1.9.4.^62^, pointed to a need of 22 subjects to achieve 80% power, at a 5% alpha in a repeated measures ANOVA, to detect effects of emotion (3 categories). We further aimed our sample at 38 participants (and used a final N = 28), following the literature’s high standard for affective research on pupil size^27,28,60,63–65^. A sensitivity analysis indicates our sample (N = 28) could detect (80% power, 5% alpha): the main effect of emotion on pupil dilation at a minimum effect size of *η*_*p*_^2^ = 0.16.

### Significance statement

For the first time, we probed authenticity recognition in human vocalizations for its effect on pupil dilation, a psychophysiological index for mental effort and arousal. We show that authentic cries and acted laughs elicited higher pupil dilation compared to acted cries and authentic laughs, respectively. These unprecedented findings suggest the socially complex process of authenticity recognition in nonverbal vocalizations can be reflected in a peripheral autonomic nervous system response, and that this effect depends on the emotion underlying the expression.

## Results

### Behavioral analysis

#### Authenticity rating

Interparticipant reliability was high for the authenticity rating (Cronbach’s α = 0.92). Expectedly, authentic vocalizations (M = 3.39, SD = 0.23) were perceived as more authentic than acted (M = 4.46, SD = 0.20) ones [*F* (1, 27) = 43.84, *p* < 0.001, *η*_*p*_^2^ = 0.62]. The effect of emotion was smaller but also significant, whereby laughs (M = 3.51, SD = 0.20) were reported as more authentic than cries (M = 4.36, SD = 0.21) [*F* (1, 27) = 16.61, *p* < 0.001, *η*_*p*_^2^ = 0.38]. No significant interaction was found (*p* = 0.670).

Participants recognized the authenticity of laughs and cries at a level that statistically significantly exceeded chance [laughter: χ^2^(1) = 145.25, *p* < 0.001 ; cry: χ^2^(1) = 152.21, *p* < 0.001], with accuracies of 63.4% and 69.3% for laughter and crying, respectively. Authenticity discrimination indexes were tested for correlation with trait empathy scores, as reported in supplemental material (Supplementary Table S2), with no statistically significant association found.

#### Arousal rating

Interparticipant reliability was high for the arousal rating (α = 0.88). Authentic vocalizations (M = 4.10, SD = 0.96) were perceived as more arousing than acted (M = 3.03, SD = 0.80) ones [*F* (1, 22) = 55.82, *p* < 0.001, *η*_*p*_^2^ = 0.72]; and laughs (M = 4.13, SD = 0.90) were perceived as more arousing than cries (M = 3.00, SD = 0.95) [*F* (1,22) = 38.22, *p* < 0.001, *η*_*p*_^2^ = 0.64]. There was also a significant authenticity by emotion interaction on arousal ratings [*F* (1.22) = 15.05, *p* = 0.001, *η*_*p*_^2^ = 0.43] (whereby the difference within laughs [t(22) = 6.56, *p* < 0.001, *η*_*p*_^2^ = 0.66], was slightly larger than the difference between cries [t(22) = 6.18, *p* < 0.001, *η*_*p*_^2^ = 0.63]).

#### Emotional contagion rating

Interparticipant reliability was also high for emotional contagion rating (α = 0.86). Authentic vocalizations (M = 4.38, SD = 0.82) were perceived as more contagious than acted (M = 3.38, SD = 0.75) ones [*F* (1,25) = 70.29, *p* < 0.001, *η*_*p*_^2^ = 0.74]; and laughs (M = 4.21, SD = 0.82) were perceived as more contagious than cries (M = 3.50, SD = 0.86) [*F* (1,25) = 21.70, *p* < 0.001, *η*_*p*_^2^ = 0.47]. No authenticity by emotion interaction effect was found (p > 0.05).

#### Correlations between ratings

Repeated measures correlation analysis indicated that the authenticity rating was positively associated with the arousal [*r*_*rm*_ (1603) = 0.42, *p* < 0.001], and the contagion ratings [*r*_*rm*_ (1460) = 0.31, *p* < 0.001]; as well as arousal and contagion ratings between them [*r*_*rm*_ (1260) = 0.40, *p* < 0.001].

### Pupil size analysis

#### Overview

**Figure 1:**
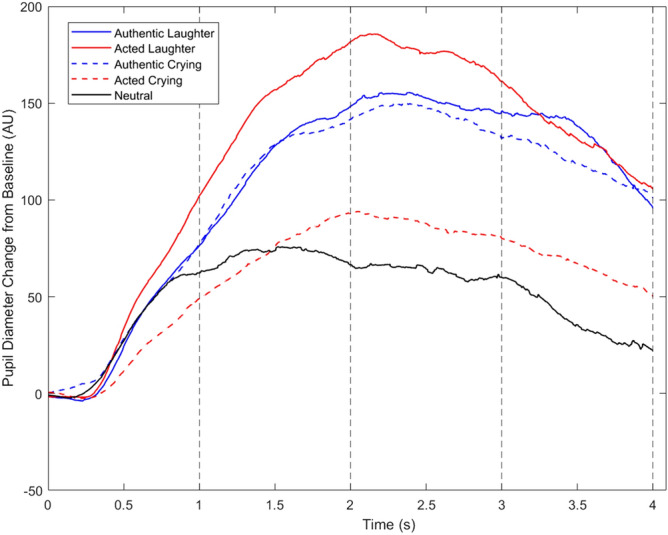
Pupil diameter change relative to baseline (in arbitrary units (AU)) for each condition, averaged across all participants. Dashed vertical lines delineate the time windows used for analysis.

MCAR tests indicated that missing values were random for the maximum and mean pupil sizes for all time windows (*p* > 0.999 for all tests). Figure 1 illustrates the pupil diameter for each condition averaged across all participants. As the main effect of authenticity on pupil dilation measures was not statistically significant (*p* > 0.05), and we verified a statistically significant cross-over authenticity by emotion interaction pattern, we will only report and discuss the latter and not the former.

#### Maximum pupil size

**Figure 2:**
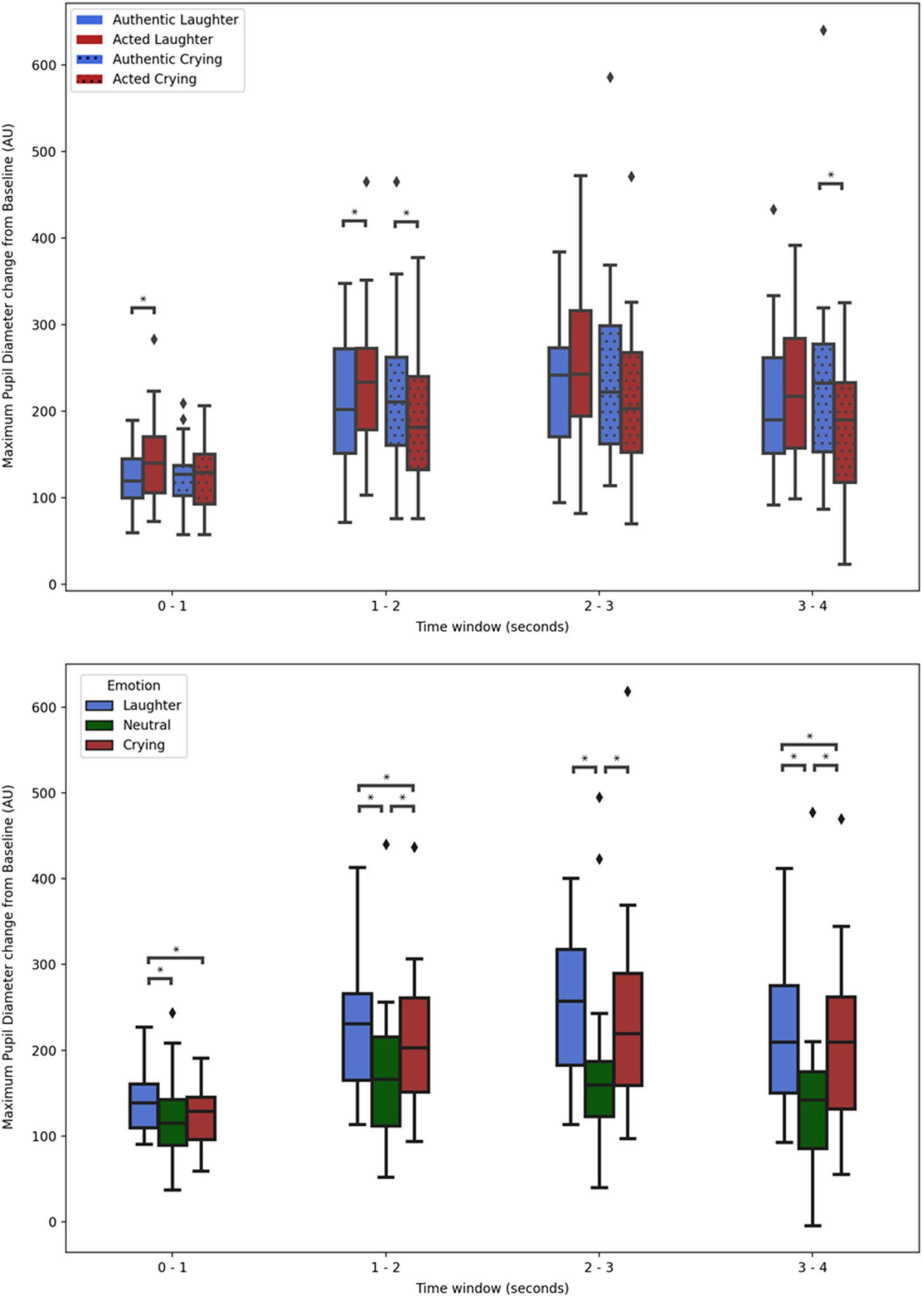
Box plots of maximum pupil diameter as a function of authenticity per emotion, showcasing the authenticity by emotion interaction (above), and of maximum pupil dilation as a function of emotion showcasing the main effect of emotion (below)—both for each time window (seconds). Statistically significant (*p* < .05, after FDR correction) pairwise comparisons are highlighted with an *. Error bars represent ± 1.5 SD; AU = arbitrary units.

The effect of authenticity on maximum pupil size was dependent on emotion (i.e. reflected in a statistically significant ‘authenticity x emotion’ interaction) [F (1, 27) = 10.60, FDR corrected *p* = 0.003, *η*_*p*_^2^ = 0.28] and, to a lower degree, on time window [F (2.32, 62.51) = 4.56, FDR corrected *p* = 0.010, *η*_*p*_^2^ = 0.15]. Pairwise comparisons for the 'emotion x authenticity’ interaction show that acted laughs (M = 216.22, SD = 12.24) elicited significantly (FDR corrected *p* = 0.028) higher pupil dilation than authentic ones (M = 192.88, SD = 10.88), and authentic cries (M = 200.91, SD = 9.92) elicited significantly (FDR corrected *p* = 0.013) higher pupil dilation than acted ones (M = 175.87, SD = 11.79), across time windows. This interaction did not significantly differ between time windows, as indicated by a non-statistically significant 3-way interaction [F (1.64, 44.12) = 1.59, FDR corrected *p* = 0.332, *η*_*p*_^2^ = 0.05]). Nevertheless, for the purpose of aiding future studies in selecting time windows for pupil size analysis, we report the ‘authenticity x emotion’ interaction results in each time window—which were all statistically significant (FDR corrected *p* < 0.05)—in the Table 1 and in Fig. 2.

**Table 1:** F-statistic (numerator and denominator degrees of freedom), *p*-value after multiple comparison correction with False Discovery Rate (FDR) and partial eta squared (*η*_*p*_^2^) for the authenticity by emotion interaction on maximum and mean pupil size, in each time window, are presented.

Time window (seconds)	Pupil size measure	Authenticity × emotion interaction			Pairwise comparisons (authentic vs. acted)						
		F-statistic (1, 27)	FDR corrected *p*-value	*η*_*p*_^2^	Emotion	M_diff_	SD_diff_	t-test (27)	FDR corrected *p*-value	*η*_*p*_^2^	Significant contrasts
0–1	Maximum	5.77	.047*	.18	Laughter	− 25.10	41.42	− 3.23	.006*	.28	Acted > Authentic
					Crying	0.47	31.38	0.08	.937	< .01	–
	Mean	4.12	.052	.13	Laughter	− 12.45	29.11	− 2.26	.064	.16	–
					Crying	6.33	28.42	1.18	.249	.05	–
1–2	Maximum	9.36	.005*	.26	Laughter	− 27.03	67.86	− 2.11	.044*	.14	Acted > Authentic
					Crying	24.98	45.42	2.91	.014*	.24	Authentic > Acted
	Mean	13.82	.002*	.34	Laughter	− 33.36	55.36	− 3.19	.007*	.27	Acted > Authentic
					Crying	18.59	46.18	2.13	.042*	.14	Authentic > Acted
2–3	Maximum	6.61	.032*	.20	Laughter	− 24.33	75.27	− 1.71	.099	.10	–
					Crying	25.33	56.78	2.36	.051	.17	–
	Mean	2.75	.109	.09	Laughter	− 14.91	85.07	− 0.93	.362	.03	–
					Cryer	21.69	56.28	2.04	.103	.13	–
3–4	Maximum	6.79	.019*	.20	Laughter	− 16.90	80.61	− 1.11	.277	.04	–
					Crying	49.36	85.42	3.06	.010*	.26	Authentic > Acted
	Mean	6.23	.019*	.19	Laughter	4.42	75.92	0.31	.760	< .01	–
					Crying	52.26	86.27	3.21	.007*	.28	Authentic > Acted

Mean difference (M_diff_), standard error difference (SD_diff_), t-test (and degree of freedom), *p*-value after FDR correction and *η*_*p*_^2^ are also presented for the follow-up post-hoc pairwise comparisons whereby authentic versus acted vocalizations were contrasted for each emotion (whilst means and SD per condition are provided in the Results’ section text).

Statistical significance level: * *p*  < .05.

Tested in a separate ANOVA including neutral vocalizations, the main effect of emotion on maximum pupil size reached statistical significance [F (1.56, 42.00) = 21.11, FDR corrected *p* < 0.001, *η*_*p*_^2^ = 0.44] as did its interaction with time window [F (2.45, 66.11) = 6.11, FDR corrected *p* = 0.001, *η*_*p*_^2^ = 0.19]. Pairwise comparisons show laughs (M = 210.77, SD = 11.62) elicited significantly (FDR corrected *p* = 0.015) higher maximum pupil dilation than cries (M = 191.98, SD = 13.69), and each higher than neutral (M = 149.26, SD = 13.90) (FDR corrected *p* < 0.001 and *p* = 0.002, respectively). In each time window, these comparisons hold statistical significance except in 0–1 s, between cries and neutral, and in the 2–3 s, between laughs and cries, as shown in Fig. 2.

#### Mean pupil size

**Figure 3:**
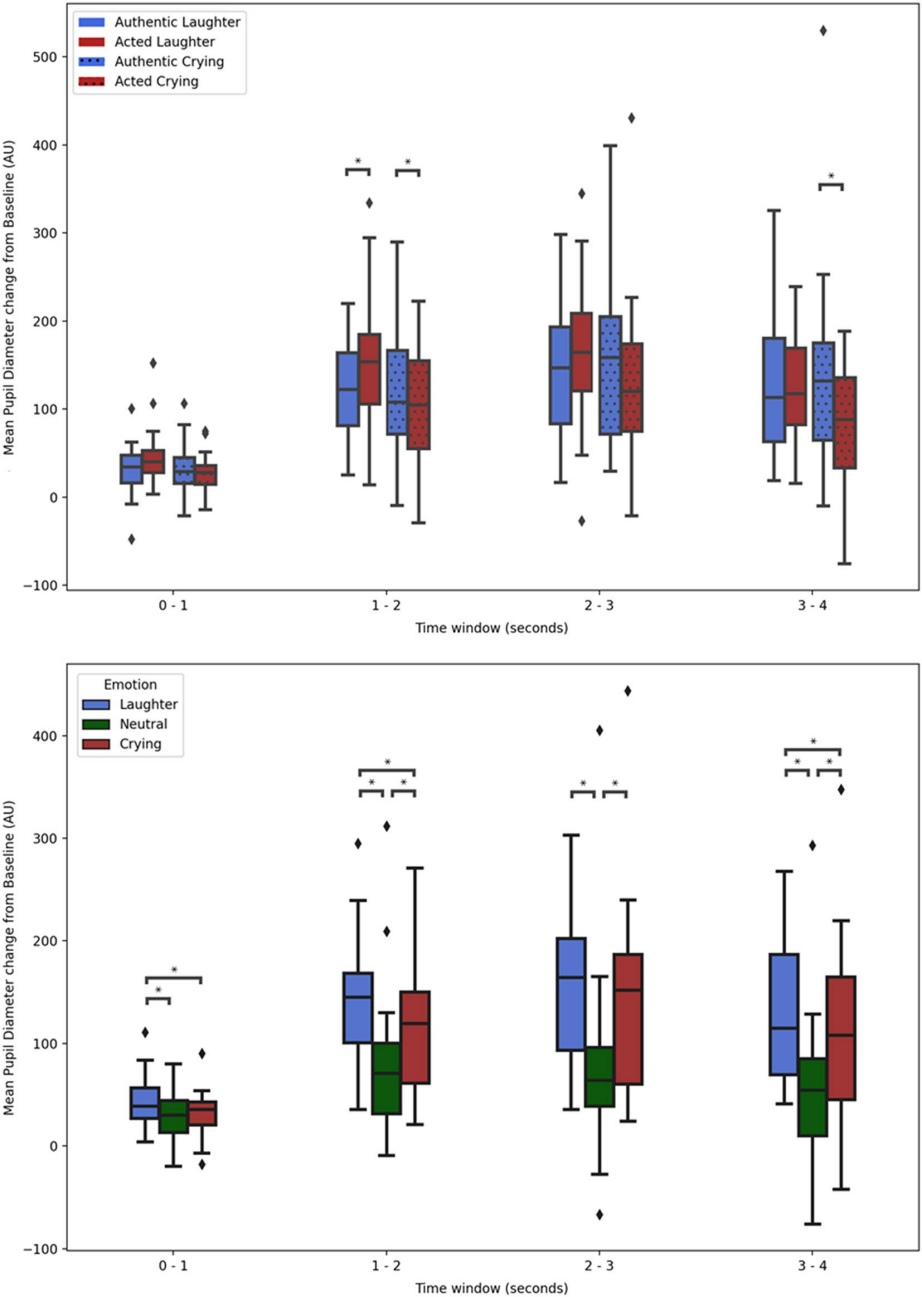
Box plots of mean pupil diameter as a function of authenticity per emotion, showcasing the authenticity by emotion interaction (above) and of mean pupil dilation as a function of emotion showcasing the main effect of emotion (below)—both for each time window (seconds). Statistically significant (*p* < .05, after FDR correction) pairwise comparisons are highlighted with an *. Error bars represent ± 1.5 SD; AU = Arbitrary Units.

The effect of authenticity on mean pupil size significantly depended on emotion [F (1, 27) = 11.15, FDR corrected *p* = 0.003, *η*_*p*_^2^ = 0.29] and on time window [F (1.80, 48.54) = 7.12, FDR corrected *p* = 0.005, *η*_*p*_^2^ = 0.21]. As on maximum pupil size, and across time windows, authentic cries (M = 109.85, SD = 12.61) elicited significantly (FDR corrected *p* = 0.013) higher mean pupil dilation than acted ones (M = 85.13, SD = 9.95), but unlike for maximum dilation, the difference between acted laughs (M = 122.68, SD = 9.32) and authentic ones (M = 108.60, SD = 9.92) did not reach significance (FDR corrected *p* = 0.114). As on maximum pupil size, this interaction did not differ between time windows, as suggested by a non-statistically significant 3-way interaction [F (1.76, 47.60) = 1.11, FDR corrected *p* = 0.332, *η*_*p*_^2^ = 0.04]). Nevertheless, we report the ‘authenticity x emotion’ interaction results in each time window—which only reached significance (FDR corrected *p* < 0.05) in time windows 1–2 s and 3–4 s—in Table 1 and in Fig. 3.

Tested in a separate ANOVA including neutral vocalizations, the main effect of emotion on mean pupil size reached statistical significance [F (1.48, 40.02) = 22.63, FDR corrected *p* < 0.001, *η*_*p*_^2^ = 0.46] as did its interaction with time window [F (2.45, 66.21) = 9.28, FDR corrected *p* < 0.001, *η*_*p*_^2^ = 0.26]. Like for maximum pupil size, pairwise comparisons show laughs (M = 117.56, SD = 9.53) elicited significantly (FDR corrected *p* = 0.006) higher mean pupil dilation compared to cries (M = 99.32, SD = 11.00), and each compared to neutral (M = 57.69, SD = 10.41) (FDR corrected *p* < 0.001 and *p* = 0.001, respectively). Also as for maximum pupil size, in each time window, the comparisons hold statistical significance except in 0–1 s between cries and neutral, and in 2–3 s between laughs and cries, as shown in Fig. 3.

#### Association of pupil size with trait empathy scores

There was a statistically significant moderate negative association between mean pupil size and the EQ Cognitive Empathy trait score in laughter [r(26) = − 0.42, *p* = 0.028]; see Table 2, but not cries (collapsing all time windows; with no statistical significance reached when considering separate windows). Authenticity was not found to significantly moderate the association between trait scores and pupil size measures in laughs and cries, irrespective of time-window (uncorrected *p* > 0.05) (Supplementary Table S3, nor within each time window (data not shown)).

**Table 2:** Pearson’s *r* and *p*-values for the correlations between pupil size measures (collapsed across all time windows) and trait empathy scores (Empathy Quotient, and its subscales, and reading the Mind in the Eyes Test scores), in each emotion separately.

Empathy trait scores	Laughter				Crying			
	Maximum		Mean		Maximum		Mean	
	Pearson’s *r*	*p*-value	Pearson’s *r*	*p*-value	Pearson’s *r*	*p*-value	Pearson’s *r*	*p*-value
Empathy quotient (EQ; total)	.28	.151	.12	.543	.22	.258	.08	.677
EQ cognitive empathy	− .37	.050	− .42	.028*	− .27	.157	− .20	.297
EQ social skills	− .14	.467	− .05	.791	− .08	.655	.18	.371
EQ emotional reactivity	− .17	.379	.04	.835	− .15	.450	.21	.290
EQ empathic difficulty	.29	.130	.11	.590	.27	.170	.05	.784
Reading the mind in the eyes test (RMET)	− .11	.567	− .30	.125	− .07	.718	− .22	.265

Statistical significance level: * *p* < .05.

#### Behavioral and pupil size analysis

None of the behavioral measures (authenticity, arousal, emotional contagion ratings) were significantly correlated (*p* > 0.05) with pupil size measures (within all time windows collapsed) which is reported as supplemental material (Supplementary Table S4). [This is in replication of an independent analysis in an independent and equally sized UK sample (data not shown or published)].

## Discussion

We asked whether the evaluation of the authenticity behind others emotional expressions might engage the autonomic nervous system. We did this by investigating whether the level of authenticity of an emotional expression affects the autonomic nervous system of the person during perception. In particular, we estimated the effect of nonverbal vocalizations’ authenticity on the pupil size of the listener. Additionally, we tested whether pupil size response depends on the valence of the emotion being vocalized—which we found to be the case. We show that a listener’s pupil size is affected by a laughter’s and cry’s authenticity such that acted laughs induce more pupil dilation than authentic ones, and, reversely, authentic cries induce more pupil dilation than acted ones. These findings are not confounded by intrinsic natural differences in the acoustic properties of authentic and acted vocalizations. Additionally, we show that laughter was perceived as more authentic, arousing and emotionally contagious than crying.

In detail, the interaction between authenticity and emotion explained almost one third of the variance in pupil dilation (i.e. 28% and 29% for maximum and mean pupil dilation, respectively), left unexplained by the other modelled main effects and interactions, including time window. Broken down per emotion, laughter authenticity negatively explained a large portion (up to 28%) of the variance unexplained otherwise, with the peak effect at 0–1 s for maximum pupil dilation, and at 1–2 s for mean pupil dilation. In crying, authenticity positively explained (i.e. in the opposite direction to laughs), an equally large portion (up to 28%) of the otherwise unexplained variance with the peak effect at 1–2 s for maximum pupil dilation, and at 3–4 s for mean pupil dilation. Even though this ‘authenticity x emotion’ interaction was present in all time windows (at least for maximum pupil size), it was most statistically significant in the 1–2 s time window (for both pupil measures, FDR corrected *p* = 0.002–0.005). In this time window, moreover, the simple authenticity effects peaked for *both* laughter and crying (and in both pupil measurements) and were all statistically significant (Table 1).

Aiming to disentangle whether it is cognitive effort (supposedly higher in acted vocalizations) or arousal (supposedly higher in authentic vocalizations) that engages the autonomic nervous system the most during authenticity recognition, since both have been associated with pupil dilation^27,66^, our results seem to support the former prediction for laughter and the latter for crying. The cognitive effort interpretation of the laughter finding is consistent with: (1) acted (vs. authentic) laughs (and their subjective discrimination) having been found to increase engagement of prefrontal cognitive empathy-relevant brain areas^1,18^, suggesting higher cognitive demand; (2) in the present study, only in laughter, pupil size was negatively correlated with the cognitive—and not the emotional—empathy score, and (3) authenticity discrimination in facial stimuli increasing with cognitive empathy trait scores^22^ (albeit, in vocal stimuli, we have only found it to increase with emotional empathy, previously^10^). The arousal interpretation of the crying finding is supported by: (1) authentic vocalizations having been found to be more arousing^18,19^, which we also replicated in the current work albeit also for laughter; (2) authentic cries being rated as much more arousing than acted ones in the current study, albeit also for laughter; and (3) crying eliciting higher amygdala activation than laughter, a highly replicated finding^67,68^, and amygdala activation being robustly and positively associated with arousal^69,70^. Thus, a possible explanation to the large and opposite effect of authenticity in laughs and cries may be that: the heightened sympathetic autonomic nervous system response we detected for acted laughs (vs. authentic) and authentic cries (vs. acted) may be due to them eliciting higher cognitive load and arousal, respectively.

Indeed solving ambiguity is cognitively demanding^71^. As indexed by pupil dilation, there is evidence that more effort is required to solve the cognitive conflict caused by auditory incongruent stimuli, compared to congruent^72^. Thus, the inherently awkward and incongruent acted vocalizations are suggestively more cognitively demanding than authentic. Moreover, previous studies show that higher cognitive demand and low confidence in emotion recognition lowers the perceiver’s discrimination ability and leads to increased pupil response^30^. We posit that the reason why we found an opposite effect for laughs and cries, may be that the discriminating authenticity in laughs depends relatively more on cognitive effort (than on emotional arousal), whilst in cries, the discrimination of authenticity may depend more on the level of emotional arousal they elicit. The hypothesis that discriminating authenticity in laughs is more cognitively demanding than in cries is consistent with it recruiting higher-order prefrontal cortical brain areas (as we have shown^1,18^), whilst a lower-order activation centered in the amygdala is typically the brain response to crying^67,68^ (although neuroimaging inspection of authenticity recognition in cries has not yet been reported). This line of thought is also consistent with the degree of ‘malignancy’ of a fake laugh and of a fake cry in social interactions. While a fake laugh is considered a more recent cultural tool^17^ to communicate polite appreciation or sarcasm, fake cries are thought to have a manipulative role. In fact, pretending criers are deemed more manipulative, less reliable, warm and competent^73^. Believing in fake cries (and then spending resources altruistically) can be costly to the person being deceived. The costlier the expenditure, the more hard-wired (and evolutionarily older) the relevant stimulus processing may be in sub-cortical brain structures; and as such the recognition of authenticity in a cry may more plausibly depend on an more lower-order amygdala-mediated emotional arousal response, more than a higher-order prefrontal-cognitive one. Indeed, crying is a biological siren and is extremely arousing for listeners as it is one of the most primitive and early behaviors we have^74^. Unlike laughter, it is the first newborn’s form of communication and arguably the most essential for their survival. This early biological underpin to cries is perhaps sufficient to facilitate our faster and more immediate authenticity discrimination in them (compared to laughter) —we may be ‘programmed’ to act urgently upon cries which are authentic, especially of newborns and children, and simply feel desensitized/unconvinced by acted cries, by perceiving them as much less arousing. Indeed, cries are arguably harder to fake and thus acted cries may be easier to spot (compared to laughs; 63% vs. 69% discrimination index accuracy in the present data), making them less cognitive demanding. Last but not least, in the present data, the acted crying was the condition rated as the least arousing to the subjects, and eliciting the lowest pupil dilation (Fig. 1), en par with neutral vocalizations which had obligatorily a minimal contribution from cognitive load due to the authenticity rating not being asked in those trials. In sum, pupil dilation displays a cumulative contribution from both arousal and cognitive effort^30^ and herein, the pupil dilated more in laughs than it did in cries (Fig. 1), suggesting that this additive effect is indeed less predominant in the latter, showcasing the presumed lower need of cognitive effort in cries’ recognition.

Regarding empathy traits, cognitive empathy is usually referred to as the ability to take the perspective of another and to understand another’s feelings or internal state, while not necessarily having an emotional response^75^. Cognitive empathy is related to social awareness and demands more cognitive effort than the more authentic emotional empathic responses, in line with its higher recruitment of the amPFC^76^. Such higher-order mentalizing computation is suggestively performed in order to distinguish between authentic and acted expressions^1,18^. Thus, in our study and, noticeably only in the laugh condition, mean pupil size was negatively correlated with the cognitive empathy score, and not correlated with emotional empathy (Table 2). This is consistent with the above deduction that non-authentic laughs triggered higher pupil dilation (vs. authentic) due to them being more cognitively difficult to decipher. If pupil size depends on cognitive load (and recruits prefrontal and cognitive empathic-relevant brain regions^31,32^, it follows that participants with superior cognitive empathy would recognize authenticity more easily, and thus show lower pupil dilation, compared to those with lower cognitive empathy scores. Finally, our reported main effect of emotion valence on pupil dilation, whereby the pupil dilated more for laughs compared to cries and each more compared to neutral, adds a piece to the puzzle to a literature context where it is currently unclear if positive or negative sounds elicit higher pupil dilation, as recently reviewed^28^. The inconsistency thus far is possibly due to the heterogeneity of the stimuli libraries being used, which could vary in volume and acoustic properties.

Regarding our behavioral findings, as expected, and consistent with previous validation of the stimuli^10,33^, participants perceived authentic vocalizations as being more authentic than acted ones, and such discrimination was statistically significant above chance level, at an 66% average in line with previous studies (ranging from 65 to 72%^15,17,19^). This indicates that our participants could correctly perceive the authenticity of the stimuli and that they were engaged in the task. Regarding the authenticity discrimination index association with emotional empathy trait scores, likely due to low statistical power, as we found the same effect direction, we could not corroborate our previous findings (with a larger sample of 119)^10^.

In respect to the effect of authenticity in all three ratings (perceived authenticity, arousal and emotional contagion ratings), it was statistically significant and these were all positively correlated with each other. As authentic vocalizations may be produced in a more spontaneous fashion (vs. acted ones), free of intention and voluntary control over the voice, they provoke higher arousal perception^15,19^. The positive correlation between ratings of authenticity and arousal, suggest that arousal perception might be involved in authenticity discrimination^19^, however conflicting associations have been reported^15,33^. Participants also rated authentic vocalizations as more emotionally contagious than acted ones, replicating our previous results^10^, and asserting the plausibility of mimicry/synchronization behaviors (e.g. body gestures, facial expressions), which are associated with emotional contagion^31^, to occur preferably when the receiver perceives emotions as authentic.

Although negative emotions have been used in authenticity perception studies^19^, the main effect of emotion (cries and laughs) on perceived authenticity, arousal and emotional contagion ratings is reported here for the first time. We report that laughs were judged significantly more authentic, arousing and contagious than cries. These differences in the perception of laughs and cries may be due to the social use of both, at least nowadays. Laughter is usually used as a “social glue” to foster agreement and cooperation and is a common form of communication between people^13^, whilst cries are usually expressed to much smaller, rarer and intimate audiences. As such, laughs may have been found more acceptable and less awkward when listened repeatedly and without context, as in such a controlled environment setting, whereas cries may have sounded more unpleasant, stranger, and thus less contagious, authentic or arousing. Our results are in line with our previous one of laughter showing to be more arousing than crying (although not statistically tested then)^33^, albeit another study found no significant difference^27^. Finally, the highly contagious effect of laughter which we found is extensively reported in the literature^10,13,77,78^.

### Potential limitations

The MCAR test^58^ was used to validate pupil size recordings^79^, and in our study, the results indicate that missing datapoints due to blinks and other recording artifacts were completely random. The analysis of the effects of authenticity and emotion on pupil size measures was divided in time windows to better characterize the effects observed, however, we do not discuss the latency of such effects as pupil size has a variable response latency^42^ and the stimuli used are continuous.

As for our complementary analyses, the fact that we did not find associations between pupil size measures and the ratings of authenticity, arousal and emotional contagion to be statistically significant is possibly because: (1) except authenticity, these were performed post-hoc after the pupillometry recording, (2) arousal was not modelled as a task condition (like all previous studies finding its association with pupil size^23,27,28^, and (3) the sample may have been insufficiently sized to examine individual behavioural differences such as arousal and contagion ratings (as it was designed to be powered to detect the pupil dilation response to the task^23,27,28,30,80^.

We acknowledge there are two additional tests that would have been useful to further support (or not) our suggestion that acted laughs may entail higher cognitive effort than authentic ones which we posed as an explanation of our present findings. First, we could have tested whether the pupil dilates more during incorrect versus correct trials for laughter, which, if so, would have supported our latter suggestion. However, the ratio of correct/incorrect trials laughs was quite unbalanced between authentic and acted laughs, which prevented a reliable statistical inference. [Specifically, for authentic laughs, participants had the double of correct trials (M = 18.61, SD = 8.62) vs. incorrect trials (M = 9.71, SD = 7.77). For acted laughs it is the reverse—on average less correct trials (M = 12.52, SD = 7.59) than incorrect (M = 15.43, SD = 0.30). Thus, considering incorrectly rated authentic laughs, multiple participants have less than 5 trials, whereas in correct authentic laughs multiple have close to 36 (the maximum).] This could however be achieved with an adapted paradigm design suitable for such question, in a future study. Second, given the nature of the authenticity discrimination index which is a score of the participant’s authenticity detection abilities for a specific emotion (calculated as described in Methods), it was also not possible to separately calculate and then compare the discrimination index for acted laughs and for authentic laughs. In a future study, an alternative index that would serve to compare the performance of authentic laughs vs acted laughs might be useful to further validate the suggested increase in cognitive effort stemming from an acted laugh.

We are aware that the exposure times to the auditory stimuli were rather short, and there was no visual information. Consequently, it was not possible to detect nonverbal emotional leakage, which made our task of distinguishing between authentic and acted nonverbal vocalizations more difficult than it is in real life. Furthermore, as pupil is a sensitive autonomic index, the recording of other autonomic indexes such as cardiac activity (e.g., Heart Rate Variability) could contribute to disentangle the influence of cognitive and affective processes in pupillary activity^81^.

The stimuli set used has different acoustic properties across conditions as detailed in supplemental material (Supplementary Table S1). For example, in the crying condition, authentic stimuli are longer than acted, and it is arguable that participants’ ratings of authenticity, arousal and contagion were influenced by their difference. When building the stimuli set, we selected excerpts from actors’ recordings (that became the stimuli used here) such that their acoustic properties were balanced as much as possible, but ultimately, we did not to control for them because we needed to preserve the natural characteristics that may make up the authenticity of a vocalization. However, we report in supplemental material that the effects of authenticity and emotion on pupil size measures we report are not attributed to differences in acoustic properties across conditions.

## Conclusions

In this work we asked if the process of authenticity recognition in nonverbal emotional cues induces an autonomic nervous system response in the listener. To do so we measured the pupil dilation of participants while exposing them to authentic and acted laughs and cries, in a task that required them to rate the authenticity of the stimuli. We report that acted laughs elicited higher pupil dilation than authentic, putatively through demanding higher cognitive effort; and that authentic cries elicited higher pupil dilation than acted ones, putatively through eliciting higher emotional arousal—in what is the first demonstration of a reflection of authenticity recognition in the autonomic sympathetic system. We also observed that authentic sounds were rated as more authentic, arousing, and contagious than acted ones, and that authenticity discrimination increases with cognitive trait empathy. Together, these findings seem consistent with available neuroimaging, psychological, cultural, and sociological features of laughter and crying. However, given their novelty, further independent examinations of the effect of others’ non-verbal vocalizations authenticity on pupil size response is warranted to validate our interpretations.

## Supplementary Information

Supplementary Information 1.

## Data Availability

The datasets generated and/or analysed during the current study are available from the corresponding author on request.
