# Supplementary material for: Pupil dilation reflects the authenticity of received nonverbal vocalizations
Source: Sci Rep. 2021 Feb 12;11:3733. doi: 10.1038/s41598-021-83070-x (PMC7880996; doi:10.1038/s41598-021-83070-x)
Supplement: Supplementary file 1 — Supplementary Information 1. [file 41598_2021_83070_MOESM1_ESM.docx]

**Supplementary Information**

**Pupil dilation reflects the authenticity of received nonverbal vocalizations**

Gonçalo Cosme^1^, MSc, Pedro J. Rosa^2,3^, PhD, César F. Lima^3^, PhD, Vânia Tavares^1,4^, MSc, Sophie Scott^5^, PhD, Sinead Chen^6^, PhD, Thomas D.W. Wilcockson^7,8^, PhD, Trevor J. Crawford^7^, PhD, Diana Prata*^1,3,9^ PhD

**Corresponding author:** Dr Diana Prata Email: [diana.prata@kcl.ac.uk](mailto:diana.prata@kcl.ac.uk).

**Supplementary Table S1**. Acoustic properties of the vocalizations: Duration (ms), mean fundamental frequency (F(0)) and mean intensity (dB).

|  | | Vocalizations’ acoustic properties | | |
| --- | --- | --- | --- | --- |
| Stimulus type | | **Duration (ms)**  **Mean (SD)**  **[min; max]** | **Mean fundamental frequency (F(0))**  **Mean (SD)**  **[min; max]** | **Mean intensity (dB)**  **Mean (SD)**  **[min; max]** |
| Laugher | **Authentic** | 2399.94 (460.73) [1536.00; 3141.00] | 397.13 (90.62)  [233.88; 560.53] | 66.10 (.10)  [65.75; 66.20] |
|  | **Acted** | 2248.89 (400.15) [1710.00; 2903.00] | 257.84 (60.26)  [130.82; 339.91] | 66.04 (.11)  [65.77; 66.19] |
| Crying | **Authentic** | 2685.44 (289.36) [2079.00; 2993.00] | 421.38 (57.04)  [307.94; 520.52] | 63.40 (3.10)  [53.43; 68.71] |
|  | **Acted** | 2322.11 (351.48) [1959.00; 2990.00] | 368.62 (87.75)  [254.57; 502.80] | 64.64 (6.93)  [50.99; 87.29] |
| Neutral | | 2498.74 (292.08)  [2057.00, 2930.00] | 182.13 (54.01)  [111.35; 258.42] | 64.81 (0.04)  [64.73; 64.91] |

Supplementary Table S2. Pearson’s r and p-values for the correlations between the authenticity discrimination index and trait empathy scores.

| Empathy Trait Scores | Authenticity discrimination index | | | |
| --- | --- | --- | --- | --- |
|  | **Laugher** | | **Crying** | |
|  | **Pearson’s r** | ***p*-value** | **Pearson’s r** | ***p*-value** |
| Empathy Quotient (EQ; total) | .03 | .90 | .01 | .96 |
| EQ Cognitive Empathy | .15 | .43 | .07 | .74 |
| EQ Social Skills | -.08 | .67 | -.06 | .77 |
| EQ Emotional Reactivity | .05 | .79 | .20 | .30 |
| EQ Empathic Difficulty | .16 | 04 | .16 | .41 |
| Reading the Mind in the Eyes Test (RMET) | .11 | .59 | .11 | .58 |

Significance level: * p < .05; ** p < .01; *** p < .001.

Our previous findings ^1^, in which emotional and cognitive empathy traits were associated with authenticity discrimination index, were not corroborated here possibly due to its relatively smaller sample (21 participants versus 119 in Neves et al.); or due to the use of different scores to assess emotional and cognitive empathy (Emotion Contagion Scale (ECS) ^2^ and Interpersonal Reactivity Index (IRI) ^3^ in Neves et al.). In the present study, the authenticity discrimination index for laughs showed a mean and standard deviation of M = 1.07, SD = 0.87; and for cries M = 1.07, SD = 0.88.

| Empathy Trait Scores | Pupil size measures | | | |
| --- | --- | --- | --- | --- |
|  | **Maximum** | | **Mean** | |
|  | **Laughter** | | | |
|  | **Beta** | ***p*-value** | **Beta** | ***p*-value** |
| Empathy Quotient (EQ; total) | -2.37 | .542 | -1.72 | .351 |
| EQ Cognitive Empathy | 5.54 | .685 | 5.48 | .403 |
| EQ Social Skills | 2.45 | .871 | 4.91 | .500 |
| EQ Emotional Reactivity | 0.53 | .970 | 2.58 | .708 |
| EQ Empathic Difficulty | -4.03 | .717 | -2.03 | .704 |
| Reading the Mind in the Eyes Test (RMET) | -6.16 | .546 | -3.30 | .500 |
|  | **Crying** | | | |
| Empathy Quotient (EQ; total) | 0.56 | .956 | -1.48 | .320 |
| EQ Cognitive Empathy | -6.52 | .634 | -3.21 | .540 |
| EQ Social Skills | -8.73 | .565 | -4.63 | .420 |
| EQ Emotional Reactivity | -3.70 | .797 | -2.26 | .675 |
| EQ Empathic Difficulty | -1.92 | .863 | -0.82 | .845 |
| Reading the Mind in the Eyes Test (RMET) | 0.52 | .960 | 0.93 | .811 |

Supplementary Table S3. Beta coefficients and *p-*values for the regression models built to test if authenticity is a moderator variable for the association between pupil size measures (collapsed across all time windows) and trait empathy scores (Empathy Quotient, and its subscales, and the Reading the Mind in the Eyes Test scores), in each emotion separately.

Supplementary Table S4. Repeated measures r_rm_ and p-values for the correlations between the behavioral and pupil size measures.

|  | Behavioural Ratings | | | | | |
| --- | --- | --- | --- | --- | --- | --- |
| Pupil size measures | Authenticity | | Arousal | | Contagion | |
|  | **r_rm_** | ***p*-value** | **r_rm_** | ***p*-value** | **r_rm_** | ***p*-value** |
| Maximum | .04 | .136 | .04 | .199 | -.04 | .160 |
| Mean | .01 | .715 | .02 | .527 | .01 | .805 |
|  |  |  |  |  |  |  |

Significance level: * p < .05; ** p < .01; *** p < .001.

Potential Mediating Effects

As reported in the methods section, and given the nature of the sounds, there are significant differences in pitch, intensity and duration between conditions (laughs vs. cries and/or authentic vs acted stimuli). If nonverbal vocalizations’ acoustic properties are, as we have found previously ^4^, mediating the recognition of authenticity, then they should be associated with pupil size measures in the same direction as their authenticity is (**Supplementary Table S5**).

Supplementary Table S5. Repeated measures r_rm_ and p-values for the correlations between the acoustic properties of the stimuli and pupil size measures.

|  | Vocalizations’ acoustic properties | | | | | |
| --- | --- | --- | --- | --- | --- | --- |
| Pupil size measures | Pitch | | Intensity | | Duration | |
|  | **r_rm_** | ***p*-value** | **r_rm_** | ***p*-value** | **r_rm_** | ***p*-value** |
| Maximum | .08 | < .001 | .02 | .254 | .04 | .017 |
| Mean | .11 | < .001 | .02 | .128 | .05 | .001 |
|  |  |  |  |  |  |  |

Significance level: * p < .05; ** p < .01; *** p < .001.

Pitch was positively correlated with all pupil size measures, and it is associated with authenticity in laughter (only), such that authentic laughter is naturally higher pitched than acted laughter. However, the effect of authenticity in all pupil size measures is such that authentic laughter elicits less pupil dilation than acted laughter. Thus pitch does not seem to be mediating the effect of authenticity on pupil size. Regarding duration, it is positively (albeit very weakly) correlated with all pupil size measures, and with authenticity in crying (only) (such that authentic cries are longer than acted cries). However, as our reported effect on pupil size is statistically significant even in intervals finishing before the acted cries do, duration does also not seem to be mediating the effect of authenticity on pupil size. Lastly, intensity is not altogether correlated with pupil size measures and as such, it is not also a mediating effect.

**SI References**

1. Neves, L., Cordeiro, C., Scott, S. K., Castro, S. L. & Lima, C. F. High emotional contagion and empathy are associated with enhanced detection of emotional authenticity in laughter. *Q. J. Exp. Psychol.* **71**, 2355–2363 (2018).

2. Doherty, R. W. The Emotional Contagion Scale: A Measure of Individual Differences. *J. Nonverbal Behav.* **21**, 131–154 (1997).

3. Davis, M. H. Measuring individual differences in empathy: Evidence for a multidimensional approach. *J. Pers. Soc. Psychol.* **44**, 113–126 (1983).

4. Anikin, A. & Lima, C. F. Perceptual and acoustic differences between authentic and acted nonverbal emotional vocalizations. *Q. J. Exp. Psychol.* **71**, 1–21 (2017).
